# Gastroesophageal Reflux Disease: A Population Based Study

**DOI:** 10.4021/gr2009.05.1291

**Published:** 2009-05-20

**Authors:** Sylvester Nwokediuko

**Affiliations:** Gastroenterology Unit, Department of Medicine, University of Nigeria Teaching Hospital, Ituku/Ozalla, Enugu, Nigeria. Email: scnwokediuko@yahoo.com

**Keywords:** Gastroesophageal Reflux Disease, Prevalence, Coffee, Kolanut

## Abstract

**Background:**

The prevalence of gastroesophageal reflux disease varies in different parts of the world. There are no population based studies in Nigeria. The main objectives of this study were to determine the prevalence and risk factors for gastroesophageal reflux disease in a population of Nigerian medical students.

**Methods:**

The Carlsson-Dent questionnaire was administered to medical students in the clinical phase of their training at the University of Nigeria, Enugu Campus. Some putative risk factors for gastroesophageal reflux disease were also included in the questionnaire.

**Results:**

The prevalence of gastroesophageal reflux disease was 26.34%. There was an association between the use of caffeine-containing substances (coffee and kolanuts) and the prevalence of gastroesophageal reflux disease (odds ratio = 2.2 and 2.015, respectively).

**Conclusions:**

Gastroesophageal reflux disease is common among Nigerian medical students. The use of caffeine-containing substances (coffee and kolanuts) by students may have a role in the high prevalence.

## Introduction

Gastroesophageal reflux disease (GERD) is a common disorder of the upper gastrointestinal tract with an incidence rate of 10% - 38% of adults in the western population occurring at least once a week [1, 2]. The prevalence has been increasing worldwide [3]. The disease affects the patients’ quality of life [4], reduces their functional activity [5], increases the economic burden [6] and predisposes them to more serious conditions as in Barretts esophagus and esophageal adenocarcinoma [7]_._

Information relating to the relationship between race and GERD is conflicting [8, 9]. Few population based studies are available from Africa but the available data suggest that in sub-Saharan Africa, GERD and its complications are rare [9]. Most studies on GERD in Nigeria were carried out on patients referred for upper gastrointestinal endoscopy [10-13]. In GERD, the dominant complaint is typically heartburn or acid regurgitation [14]. A patient-centered, symptom-driven approach to GERD assessment and diagnosis is currently recommended and it is independent of endoscopic findings [15].

Symptom analysis is a practical and inexpensive approach to the diagnosis of GERD. It enables identification of most patients with GERD who present with typical symptoms [1, 4]. This is the rationale for the increasing use of structured questionnaires in the diagnosis of GERD. The Carlsson-Dent questionnaire [16] has widespread acceptance, as in a Chinese report for the diagnosis of GERD [17]. The contents of the questionnaire are consistent with the Montreal definition and classification of GERD as articulated in a global evidence-based consensus [15].

This population-based study aimed at determining the prevalence of GERD and risk factors associated with it among medical students in a Nigerian medical school using the Carlsson-Dent questionnaire.

## Materials and Methods

This was a cross-sectional questionnaire-based study of medical students in the clinical stage of their training at the College of Medicine, University of Nigeria, Enugu Campus. The study lasted from February 1, 2008 to April 30, 2008. Informed consent was obtained from all the participants and all consenting students were included. Students who were pregnant at the time of the study were excluded.

The Carlsson-Dent questionnaire was administered to all the participants. This is a 7-item questionnaire which utilizes a symptom description as well as symptom analysis. Numerical scores are assigned to specific components of the symptom analysis. These scores could be positive or negative. In the end the scores were summed to obtain a total score which ranged from -7 to +18. The severity of symptoms was also graded from 1 to 5 representing no problem at all, mild problem, moderate problem, severe problem and very severe problem. There were 2 conditions for a diagnosis of GERD: (1) a total score of 4 or higher on the 7-item Carlsson-Dent questionnaire [16]; (2) mild symptoms occurring on 2 or more days a week or more severe symptoms occurring at least once a week [15, 18].

Other items included in the questionnaire included consumption of alcoholic drinks, smoking, use of non steroidal anti inflammatory drugs (NSAID), consumption of coffee and consumption of kolanuts. The statistical program SPPS Version 12 was used in the analysis of results and the results were expressed as means, standard deviation, and proportions. Where appropriate, proportions were compared using Chi-square test and a P value of less than 0.05 was considered significant. Strength of associations was also expressed as relative risk and odds ratio.

## Results

Four hundred and ten (410) medical students completed the questionnaire. These were made up of 240 males (58.7%) and 170 females (41.5%). Their ages ranged from 19 years to 50 years (mean = 25.3 ± 3.5 years). The body mass index (BMI) of the students ranged between 17 and 55.12 (mean = 24.11 ± 4.50). One hundred and eighty students (26.34%) satisfied the criteria for diagnosis of GERD, i.e. score of 4 and above in the Carlsson-Dent questionnaire plus symptoms of significant severity [15, 18]. These were made up of 54 males (50%) and 54 females (50%). The difference between the proportion of male students with GERD and the proportion of female students with GERD was not statistically significant (P= 0.111). The remaining 302 students (73.66%) did not satisfy the criteria for GERD.

A test of correlation was carried out between BMI and GERD and there was no correlation (γ= 0.00471, P = 0.9252). Similarly, a test of correlation was carried out between age of the students and GERD and there was no correlation (γ= -0.01052, P = 0.8428). Smoking was recorded in 8 of the students who had GERD and in 18 of those who did not have GERD. The difference between the proportions was not statistically significant (P = 0.6203, relative risk = 1.168, odds ratio = 1.243). Alcohol consumption was noted in 48 students with GERD and in 120 students without GERD. The difference between the proportions was not statistically significant (P = 0.5834, relative risk = 1.085, odds ratio = 1.119). Use of non-steroidal anti-inflammatory drugs (NSAID) was noted in 40 GERD students and in 92 students without GERD. The difference between the proportions was also statistically not significant (P = 0.3743, relative risk = 1.15, odds ratio = 1.216). Use of coffee was noted in 59 students with GERD and 75 students without GERD. The proportion of GERD students who consumed coffee was therefore more than the non-GERD students and the difference was statistically significant (P = 0.0001, relative risk = 1.672, odds ratio = 2.2). Use of kolanut was noted in 48 GERD students and in 68 students without GERD. The proportion of GERD students who consumed kolanut was higher than that of non-GERD students and the difference was statistically significant (P = 0.0012, relative risk = 1.59, odds ratio = 2.015). Table 1 illustrates the relationship between some of the putative risk factors and GERD.

**Table 1:** Relationship between GERD and some putative risk factors

Risk factor	GERD (n = 108)	No GERD (n = 302)	P - value	Relative Risk	Odds Ratio
Smoking	8	18	0.6203	1.168	1.243
Alcohol	48	120	0.5834	1.085	1.119
NSAID	40	92	0.3743	1.15	1.216
Coffee	59	75	0.0001*	1.672	2.2
Kolanut	49	68	0.0012*	1.59	2.015

*Statistically significant

## Discussion

There is general agreement among researchers that the prevalence of GERD varies in different parts of the world. A common misperception is that GERD and its complications are rare in Africa [8, 9]. A prevalence of 26.34% among Nigerian medical students shown in this study suggests that GERD is actually common and not rare as previously thought.

The medical students who participated in the study had a mean age of 25.3 ± 3.5 years. Age did not correlate with the presence of GERD symptoms. This is different from the experience of some other researchers on this subject who documented a rising incidence of GERD with age [19]. The reason for this may be the fact that most of the students were in their third and fourth decades of life. A similar study in a more heterogeneous population with broader range of categories of age will most likely give a higher prevalence and the effect of age will be more evident.

Previous studies showed that male gender is a risk factor for erosive esophagitis; whereas females are more likely to be associated with non-erosive reflux disease (NERD) [20, 21]. In this study the prevalence of GERD in male medical students was not different from that of female students. However, it is not possible to predict whether esophageal mucosal lesions would have differed in the two groups because the study was purely questionnaire-based, no endoscopy was done.

BMI did not affect the prevalence of GERD in the students. The relationship between BMI and symptoms of GERD has remained unresolved [22-24]. The effect of BMI on GERD may have come out better if the study population had included older persons who are likely to have higher BMI.

Smoking, alcohol and NSAID use did not affect the prevalence of GERD in this study. Previous studies gave conflicting reports on the effect of smoking [25, 26]. The role of NSAID use in the etiopathogenesis of GERD is equally controversial [27, 28]. However the use of caffeine containing beverage namely coffee and kolanut was clearly more prevalent among the students who had GERD compared to those without GERD (P = 0.0001, relative risk = 1.672, odds ratio = 2.2 for coffee; and P = 0.0012, relative risk = 1.59, odds ratio = 2.015 for kolanut). Coffee drinking is not very common among the general population in Nigeria but may be prevalent among students who drink this beverage to keep awake in order to read especially during examinations. Kolanut has the same effect and is even cheaper. This nut is widely grown in the South Western part of Nigeria but extensively consumed in the Northern and South Eastern parts of the country. GERD may be prevalent in those kola-eating areas. Larger population-based studies are needed to further elucidate this view.

The use of antibiotics for the eradication of *Helicobacter pylori* (*H. pylori*) related gastroduodenal disorders became widespread in Nigeria about 15 years ago with the result that many dyspeptic patients are rightly or wrongly placed on this treatment. Drugs for the eradication of *H. pylori* are administered indiscriminately because there are no guidelines for the use of these drugs and even where the guidelines exist; they are not followed properly because of constraints of poor laboratory support. It is possible that such widespread use of drugs for *H. pylori* eradication may be attended by a rise in the prevalence of GERD and its complications [29-31]. This thinking needs to be further elucidated by research.

A questionnaire-based study such as this is not without limitations. The Carlsson-Dent questionnaire deals mainly with the major esophageal syndromes of GERD. The atypical features such as chest pain, epigastric pain, laryngitis, chronic cough and asthmatic attacks are not included. The result is that the prevalence determined in this study may be less than what actually obtains. Another limitation is the fact that symptom analysis was not compared with any confirmatory test for GERD. However, it is difficult nowadays to talk about a gold standard for the diagnosis of GERD.

Endoscopy has relatively low sensitivity as the absence of visible mucosal breaks is reported in 55% to 81% of patients presenting with reflux symptoms in primary care [32]. Ambulatory 24 hour pH monitoring is the most widely used test to establish the presence of excessive GERD, and to correlate symptoms temporally with reflux. This test is invasive, inconvenient and costly. Also there exists no absolute threshold value that reliably identifies GERD patients. Investigators evaluating the sensitivity of pH monitoring report obtaining normal esophageal acid exposure in 25% of patients with reflux esophagitis and about 30% of patients with non-erosive reflux disease [33-35].

In conclusion, GERD may not be rare in Africans as previously thought. The prevalence in Nigerian medical students is high and this may be related to the consumption of caffeine-containing substances namely coffee and kolanut consumed commonly by students in their bid to exploit the cerebral stimulant effect of these substances during examinations when they need to stay awake and read. There is a need for more population-based studies on GERD in Nigeria.
